# Frequency‐Controlled Fluidic Oscillators for Soft Robots

**DOI:** 10.1002/advs.202408879

**Published:** 2024-10-08

**Authors:** Mostafa Mousa, Ashkan Rezanejad, Benjamin Gorissen, Antonio E. Forte

**Affiliations:** ^1^ Department of Engineering King's College London Strand London WC2R2LS UK; ^2^ Department of Mechanical Engineering KU Leuven Celestijnenlaan 300 Leuven 3000 Belgium; ^3^ Flanders Make Celestijnenlaan 300 Leuven 3000 Belgium

**Keywords:** electronic‐free circuits, pneumatic control, soft robotics

## Abstract

Electronic‐free controls have recently emerged as one of the main topics in soft robotics. However, electronic‐free fluidic circuits still lack controllability and reconfigurability to achieve different functions. Here, reconfigurable pneumatic valves that widen the design space of fluidic circuits are presented. The significance of two parameters on the valve's operation: a pre‐defined manufacturing parameter that sets the initial operational range of the valve, and a second, on‐the‐fly modifiable geometric parameter that shifts the behavior of the valve during operation is shown. It is demonstrated that equipping the valve with these reconfigurable features enables the tuning of fluidic oscillatory circuits as illustrated by two examples: a frequency‐controlled relaxation oscillator and a reconfigurable ring oscillator. The relaxation oscillator is employed to control the actuation frequency of a soft hopper, which is able to achieve 80 to 125 hops min^−1^ and a hopping speed ranging from ≈1 to ≈1.185 BL s^−1^. Additionally, the reconfigurable ring oscillator is used to demonstrate how each output frequency can be controlled independently, via a soft robotic crawler that can navigate in three directions, and a volume‐controlled fluidic pump able to achieve mixing of solutions in environments where electronic components cannot operate.

## Graphical abstract

This work investigates how reconfigurability enables better control of soft robots. By embedding reconfigurable geometric parameters in fluidic valves' design, soft robots can operate in different modes without replacing circuit components or introducing new pressure inputs. This is showcased by a soft hopper that hops at different speeds, a soft crawler that locomotes in different directions, and an electronic‐free fluidic pump.

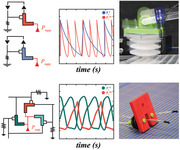

## 1 Introduction

Soft robots^[^ ^1^ ^]^ have become complementary to classic, hard robots, as they are inherently safe,^[^ ^2^, ^3^ ^]^ resilient,^[^ ^4^, ^5^ ^]^ and pliable,^[^ ^6^, ^7^ ^]^ and have enabled the design of novel wearable devices,^[^ ^8^, ^9^ ^]^ manipulators,^[^ ^10^, ^11^ ^]^ and locomotors.^[^ ^12^, ^13^, ^14^ ^]^ Controlling soft robots usually relies on traditional electro‐pneumatic systems, which involve the use of solenoid valves, regulators, pumps, and electronic controllers.^[^ ^3^ ^]^ These hinder untethered operation, and agility, and impede miniaturization. Hence, reducing or eliminating soft robots' reliance on such devices is an increasingly important task and one that is gathering significant attention in the field.^[^ ^15^ ^]^

Recently, scientists have investigated novel designs for soft valves and pumps, to replace commercially available, rigid ones.^[^ ^16^, ^17^, ^18^ ^]^ Such devices provide enhanced compatibility with soft robots and improve their capacity,^[^ ^2^ ^]^ as they can be integrated into the robot's body, reducing centralized controls, utilizing its body efficiently,^[^ ^19^ ^]^ and enabling deployment in extreme environments.^[^ ^20^ ^]^

Kinking tubes are one of the main means adopted to interrupt the fluid flow in soft valves mechanically,^[^ ^21^ ^]^ which usually harness bistability^[^ ^22^ ^]^ or buckling^[^ ^12^ ^]^ to switch between on/off states. By doing so, robots can perform tasks with a reduced number of control inputs. Examples include sensing,^[^ ^22^ ^]^ shift registers,^[^ ^23^ ^]^ and digital‐to‐analog converters.^[^ ^24^ ^]^

Control input reduction is indeed necessary for the design of compact and untethered soft robots. To this aim, a range of mechanical valves has recently been developed to enable a series of functionalities whilst keeping the number of pressure inputs at a minimum. These span from one‐way valves to bistable and hysterical designs, which can memorize or forget the last state after the trigger is removed, respectively.^[^ ^25^ ^]^

Additionally, soft valves have been used to create oscillators, enabling robots to have their own clock, similar to computers, through strategies involving one or more connected valves. These electronics‐inspired configurations allow the output of these valves to have periodic and/or sequential activation, unlocking different modes of operation,^[^ ^15^ ^]^ such as locomotion modes like swimming and climbing,^[^ ^12^ ^]^ and controlling pressure/time in lab‐on‐a‐chip devices.^[^ ^26^ ^]^ Some of the newest soft valve designs can be programmed to shift their operational pressure range.^[^ ^27^ ^]^ In a related development, scientists have recently designed pressure‐controlled oscillators by employing a series of valves analogous to PMOS and NMOS transistors.^[^ ^28^ ^]^ However, this approach requires a large number of valves and additional regulating pressure to control the oscillator frequency.

**Figure 1:**
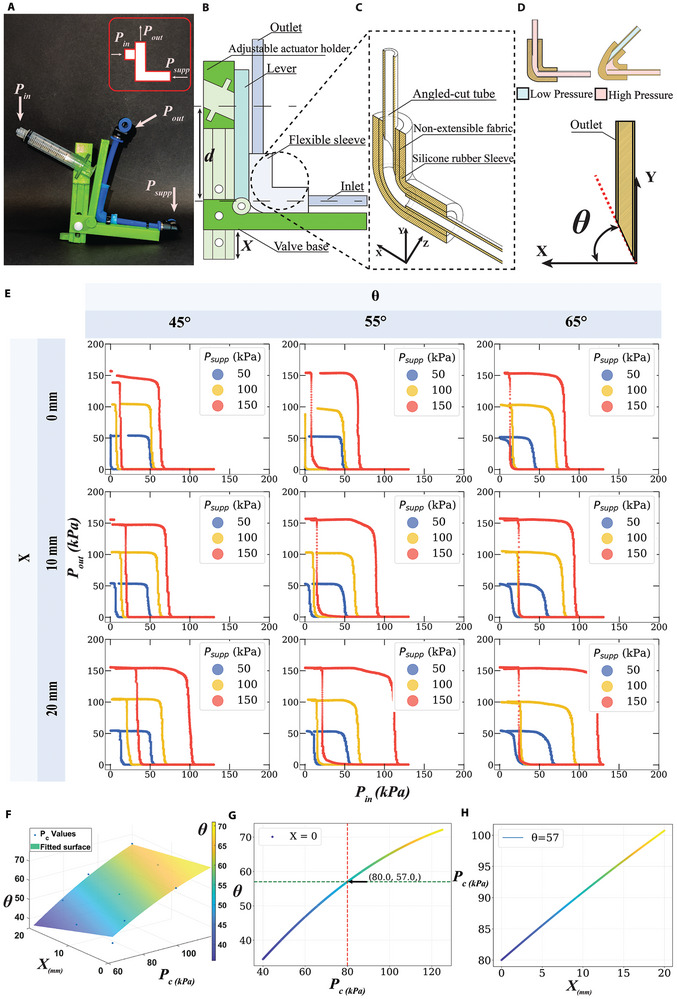
Reconfigurable valve design and characterization. The valve design, comprising, a soft sleeve, pneumatic tubes, a 3D‐printed adjustable mechanism and its symbol, which is used throughout the manuscript A). The different valve components and the reconfigurable parameter *X*, and *d* definition B). The cross section of the soft sleeve structure C). The soft sleeve's cross section in its actuated and non‐actuated forms and demonstration of the tube cutting angle θ D). The valve characterization across different supply pressures (*P*_*supp*_), θ, and *X* E). 3D fitted model for *P*_*c*_, *X* and θ at *P*_*supp*_=150 kPa F). An example of θ selection based on a desired *P*_*c*_ G) and all the possible *P*_*c*_ values when sweeping *X* for the chosen θ H).

Differently, we hereby introduce a reconfigurable valve to adjust the frequency of oscillatory fluidic circuits i) on‐the‐fly, i.e., while operating the circuit, ii) without changing the circuit's supply pressure or using an additional regulating pressure, and iii) without changing any of the circuit components whilst maintaining the circuit's pressure and flow unaltered, which is important for the safety of soft actuators. Our valve design (**Figure** **1**A) allows us to i) program its operational range and ii) change its characteristic behavior by modifying one geometrical parameter during operation. The second feature is fundamentally in contrast with electronics, where manufactured transistors have a predefined and fixed behavior. In particular, the analog control of the oscillator frequency is embodied into the valve itself ‐ in the form of a sliding actuator holder ‐ and it is achieved without changing the circuit's input, which in turn contributes to the embodiment of computation directly into robotic devices.^[^ ^29^ ^]^ The valve is based on kinking a flexible non‐extensible sleeve that connects two tubes (see Figure 1B). This flexible sleeve is manually placed in a 3D‐printed case. The case consists of two parts, a valve base, and the sliding actuator holder. A linear actuator is placed facing the lever, which when pushed kinks the sleeve. Since “soft for soft's sake” approach^[^ ^30^ ^]^ can hinder soft robotics potential, our valve utilizes a hard‐soft hybrid design that can withstand high operating pressures (3 bars). However, we also demonstrate that the valve's working principle maintains its validity even when we modify constitutive materials and components. In particular, we show that the device can be miniaturized and manufactured to be completely soft, reaching oscillation frequencies among the highest in literature.^[^ ^31^ ^]^ In this study, we define the pressure supplied at the valve inlet as *P*_*supp*_, the pressure measured at the valve outlet as *P*_*out*_, and the pressure at the linear actuator as *P*_*in*_ (see Figure 1A). Furthermore, we define the closing pressure when the valve switches from ON to OFF (and the airflow gets blocked) as *P*_*c*_ and the opening pressure when the valve switches from OFF to ON (and air can flow again from the inlet to the outlet) as *P*_*o*_.

## 2 Results

### 2.1 Reconfigurable Valve Characterization

The reconfigurable valve is based on kinking a flexible sleeve that connects two tubes. This sleeve is made of a silicone rubber cylinder reinforced with a non‐extensible fabric. The fabric prevents the sleeve from ballooning when pressurized, whilst still allowing it to bend. Two tubes are connected to the sleeve, and the one at the outlet side is cut with a chosen angle (θ). After securing the tubes to the sleeve, the assemblage is placed in a 3D‐printed case, composed of i) a valve base, ii) a lever that is free to rotate around a pin (or hinge), and iii) an adjustable actuator holder, which has a slot designed to fit a common pneumatic linear actuator (syringe, Figure 1A). The adjustable actuator holder is free to slide vertically inside the valve's base in both directions getting closer to the pin or away from it. The distance *X* is defined as the length of the actuator holder that extends beyond the valve's base (Figure 1B).

When pressure is applied to the linear actuator (*P*_*in*_) this is translated into a force that pushes the lever and the outlet tube, which in turn kinks the sleeve, and blocks the flow. Depending on the angle θ (chosen during the fabrication) and the parameter *X* (reconfigurable on‐the‐fly by sliding the holder into the base), the pressure threshold (*P*_*c*_) where the valve switches from ON to OFF can be changed. In particular, the higher the pressure *P*_*in*_ (to the linear actuator) the more force is applied *F* = *P*_*in*_ × *A*, where *A* is the surface area of the actuator's piston that the pressure *P*_*in*_ acts upon. The bending moment, *M*, can be described as *M* = *F* × *d*, where *d* is the distance from the actuator to the center‐line passing through the inlet tube cross section. Decreasing the distance *d* (i.e., increasing *X*) results in a higher force required to bend the sleeve. In other words, more pressure (*P*_*in*_) is needed to reach the bending moment *M* at which the valve switches from open to close position. This allows us to change the pressure threshold (*P*_*c*_) after the valve has been manufactured by only changing one geometrical parameter (i.e., *X*).

As mentioned, the operational pressure range of the valve can be further modified by introducing a second geometric parameter: the angle θ. This parameter can be easily set in the assembly phase and impacts the valve behavior significantly. Both θ and *X* affect the *P*_*c*_ as visible in Figure 1E. To demonstrate the available operation range of our valve, we report characterization experiments for three θ angles and three *X* offsets: 45°, 55°, and 65°, and 0, 10, and 20 mm, respectively. All combinations of angle and offsets were tested at three different *P*_*supp*_: 50, 100, 150 kPa. Additionally, we report changes in *P*_*c*_ values (defined as the value of *P*_*in*_ when *P*_*out*_ falls to 50% of the supply pressure *P*_*supp*_) in Figure S1 (Supporting Information). Further characterization experiments are carried out for different flow rates (see Figure S2, Supporting Information).

A second‐order polynomial fitting is applied to the acquired data (*P*_*c*_, X, and θ) for every *P*_*supp*_. The fitted model for *P*_*supp*_ = 150 kPa is shown in Figure 1F, which represents a guideline for choosing a suitable θ based on a desired *P*_*c*_. The model can predict all *P*_*c*_ values that can be achieved by sweeping *X* during valve operation. For instance, to build a valve that has a *P*_*c*_ = 80 kPa in a circuit where *P*_*supp*_ = 150 kPa, the model suggests choosing θ = 57° (see Figure 1G). For this θ, all possible *P*_*c*_ values when sweeping *X* are shown in Figure 1H. The fitted models for *P*_*supp*_ = 50, 100, 150 kPa are added to Supporting Information (see Figure S3, Supporting Information).

The flexible sleeve allows us to control the kinking precisely, which has an advantage. Compared to valves that harness bistability to switch state,^[^ ^22^, ^28^ ^]^ our valve shows a smooth transition between ON and OFF states. This relationship is continuous, allowing intermediate states (i.e., partially obstructed flow, around *P*_*c*_) to be selected during operation, via fine regulation of the input pressure.

Finally, the characterization experiment shows a hysteretic behavior. The valve switches OFF after *P*_*in*_ is greater than *P*_*c*_, and resets to ON when *P*_*in*_ is lower than the valve opening threshold (*P*_*o*_, quantified in the same way as *P*_*c*_). The characteristic curves show that *P*_*o*_ is significantly lower than *P*_*c*_. This hysteresis is a crucial aspect in the design of oscillatory circuits, which is the focus of this work.

### 2.2 Variable Speed Soft Hopper

**Figure 2:**
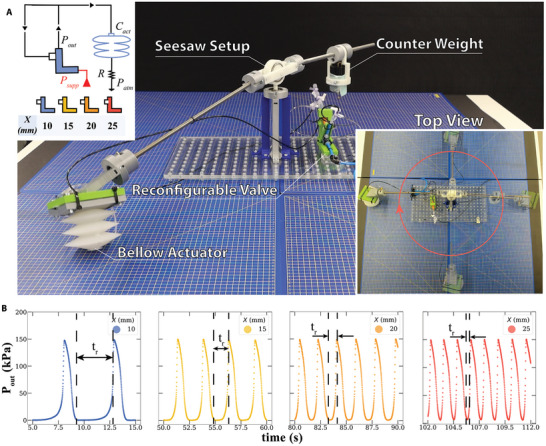
Soft hopper. Picture of the soft hopper setup; the reconfigurable relaxation oscillator circuit driving the soft hopper on the top left corner, and the top view snapshots of the hopper at different time instants on the bottom right corner A). The output pressure waveforms of a frequency‐controlled pneumatic oscillator across four different *X* values B).

To showcase the capability of our valve, we incorporate it in a relaxation‐oscillator circuit which provides a continuous periodic change between high (charging) and low (discharging) pressure states as output. During charging of the circuit shown in **Figure** **2**A, the pressurized air passes from the valve inlet to its outlet, as the valve is open (ON state). Since in this particular circuit, the outlet is connected to the valve's actuator, pressure builds up in the actuator, which starts to extend, bending the sleeve until it is completely kinked.

This is the end of the charging phase and the start of the discharging phase. After the sleeve is kinked, the trapped pressurized air escapes the circuit via a pneumatic resistor (extra tubing; 0.5 m FESTO Poly‐urethane tube 3.25 mm inner diameter, 4 mm outer diameter) between the valve's actuator and the atmosphere. After the pressurized air escapes the circuit, and the valve's actuator returns to its initial position (valve resets to open state), the charging phase starts again.

The frequency of this oscillator can be guided by many parameters, for example, different values for the circuit's capacitors and resistors. However, we here fix all parameters and investigate the effect of *X* on the oscillatory frequency (θ was kept constant at 45°). We found an inverse relationship between *X* and the valve rise time (*t*_*r*_), which is the time between the drop of *P*_*out*_ to 0 and the rise of *P*_*out*_ (see Figure 2B; Figure S4, Supporting Information). In other words, increasing *X* reduces the time the sleeve needs to relax and reset to the new charging phase. Therefore, the oscillatory frequency of our circuit can be easily controlled by externally changing the parameter *X*, as shown in Figure 2B, where we report the *P*_*out*_ oscillations as a function of time for four values of *X*.

We then use the relaxation oscillator circuit to create the first soft hopper driven by a frequency‐controlled oscillator. Hoppers are one of the most challenging designs in robotics,^[^ ^32^, ^33^ ^]^ as these systems require high response times, adaptability, and agility. Here we pair the frequency‐controlled relaxation oscillatory circuit, a soft bellow actuator, and a counterweight in a rotational seesaw mechanism (Figure 2A) to obtain a variable‐speed soft hopper.

We position the soft bellow actuator just after the valve output. This circuit discharges through the actuator itself which is connected to a tube open to the atmospheric pressure *P*_*atm*_, equivalent to the pull‐down resistors in the electronics realm. For more information regarding the soft bellow actuator, please see Figure S5 (Supporting Information).

By tuning the oscillatory pressure output provided to the soft hopper (via the parameter *X*) and the counterweight concerning the oscillating frequency, we can achieve continuous circumferential hopping. The hopper speed varies from 1 to 1.85 BL s^−1^ as can be seen in Movies S3 and S4 (Supporting Information).

### 2.3 Reconfigurable Ring Oscillator

Ring oscillators are another type of circuit that generates periodic oscillations at their outputs in both the pneumatic and electronics realms. In the case of pneumatics, ring oscillators can be set up using normally open valves (or a pair of normally open and normally closed valves). Whilst, in electronics, CMOS inverters are used to set up such circuits. By connecting any odd number of inverters in series (where each output is connected to the following inverter input), a system‐level instability arises, where no inverter can hold its ON or OFF state. When using three inverters, the circuit always has two adjacent inverters in the OFF state where one is about to switch ON, or two adjacent inverters in the ON state where one is about to switch OFF. This creates an instability that propagates with the inverters continuing to alternate between these states. The output of such a circuit is three oscillations where the ON state of each lasts for one‐third of a time period.

Fluidic ring oscillators have recently been employed to enable locomotion^[^ ^12^, ^15^ ^]^ and control strategies in soft robots.^[^ ^34^ ^]^ In current applications, the oscillating frequency is not controlled or altered once the circuit is built. However, controlling the phase and frequency of each output in a ring oscillator has the potential to enrich the available functionalities of a given system.

**Figure 3:**
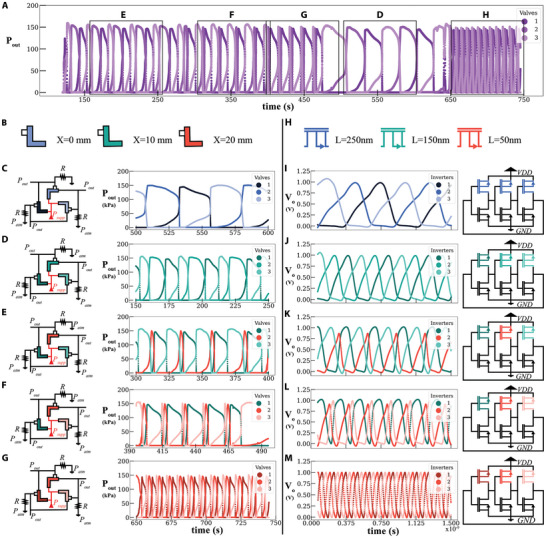
Reconfigurable ring oscillator. The oscillations of the reconfigurable ring oscillator at different valves offsets, 100 s time window is defined per configuration A). The color code of the valves is related to their *X* values, where shades of blue, green, and red are assigned to valve configurations of *X*=0, 10, and 20 mm, respectively. Different shades of the same color are used to differentiate output waveforms for valves that have the same *X* B). The fluidic circuits at different configurations and their output oscillation waveforms C–G). Different PMOS at variable lengths used in CMOS inverters are color‐coded similarly to the valves H). Equivalent CMOS ring oscillator circuits and their output waveforms I–M).

To this end, we here present a reconfigurable ring oscillator circuit comprising three reconfigurable valves. This allows changing the frequency of oscillations for every valve's output as shown in the full stream of data in **Figure** **3**A. In this circuit, the outlet of each valve is connected to the input (actuator) of the following valve. All valve inlets are connected to the supply pressure, and each valve actuator is connected to a pull‐down resistor to the atmosphere (2 m FESTO tubing with an inner diameter of 2 mm). After a short initial transient (≈2 s), instability emerges (as explained earlier) and the valves start shifting from ON to OFF states periodically.

The same approach followed in the relaxation oscillator experiment is employed here, where every parameter is fixed (resistors, and θ = 65°), and only the effect of changing *X* (0, 10, 20 mm) is investigated (see Figure 3B with different color‐coding of *X*). The results in Figure 3C,D,G show that an equal increase of *X* for all valves results in an equal shift of frequency, with each valve remaining ON for a third of a time‐period. The frequency is proportional to the *X* values for each circuit, where in Figure 3C at *X* = 0 mm, the frequency recorded is the lowest. When *X* is changed to 10 mm the frequency is increased by three‐folds (Figure 3D), same behavior is observed when *X* is changed to 20 mm (Figure 3G).

We then investigated the circuit's capability to alter one oscillation while keeping the other two oscillations unchanged. By transitioning from *X* = 10 mm (Figure 3D) to *X* = 20 mm (Figure 3G), we mismatch one valve at a time to observe the effect on the oscillations. The results in Figure 3E show that mismatching one valve reduces its ON‐period. Similarly, when another valve is changed to *X* = 20 mm (Figure 3F), both valves exhibit a reduced ON‐period, while the unchanged valve maintains its original behavior. We believe this feature is significant for soft robotics applications, as demonstrated in the upcoming sections.

Next, we look into validating the reconfigurable ring oscillator behavior by equivalent electronics simulations (using LT Spice). A basic CMOS inverter circuit consists of a PMOS and an NMOS transistor, where their gates and drains are connected. At HIGH input for the gates, the output is LOW, and vice versa. We run simulations of a CMOS ring oscillator that includes three different CMOS inverters. To induce a change in the CMOS inverter so that it is similar to our reconfigurable valve (i.e., different *X*), we change its PMOS length to have three different values: 50, 150, 250 nm (see Figure 3H). The increase in the PMOS length impacts the CMOS inverter rise time, similarly to how the parameter *X* impacts the fluidic circuits (see Figure S4, Supporting Information).

The results of the simulations of CMOS ring oscillators with identical inverters show three oscillating voltage outputs each lasting for a third of the time‐period (see Figure 3I). The frequency increases when using inverters with a PMOS of reduced length (see Figure 3J,M). Interestingly, mismatching one inverter at a time changes the output frequency of the mismatched inverter similar to the equivalent fluidic circuit (see Figure 3K,L). Importantly, we can change the *X* value of the valves on‐the‐fly but changing the PMOS length of transistors after fabrication is impossible.

### 2.4 Soft Steerable Crawler

**Figure 4:**
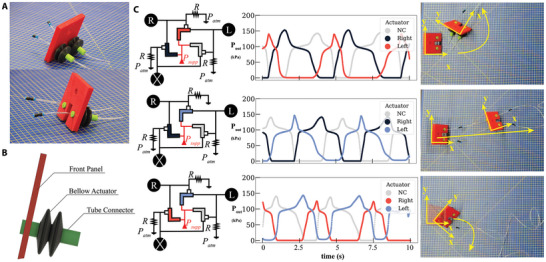
Soft Steerable Crawler Two isometric views of the soft crawler A). Schematic showing the different components of the crawler labeled B). The different ring oscillator circuit configurations and its output waveforms and the corresponding crawler motion. In the graphs, Right refers to the right‐hand side actuator, Left to the left‐hand side actuator, and NC to the not‐connected output C).

Since we now have the capability to manipulate each of the oscillatory outputs in our ring oscillator, we hereby show how this can be useful to achieve functionality. To this end, we build a soft crawler (see **Figure** **4**A) that is capable of steering. The crawler is manufactured by 3D printing silicone bellow actuators using a stereolithography printer (formlabs Silicone 40A resin on a Formlab Form 3B+). Two bellow actuators are connected to a front panel (3D printed with Ultimaker TPU‐95 on an Ultimaker S5 printer, see Figure 4B) to form the crawler. Each bellow is connected to one of the reconfigurable ring oscillator outputs (θ = 55°) (leaving the third output open to the atmosphere). By increasing *X* in one of the valves, the duration of its ON state decreases significantly. Hence, the actuator connected to this specific valve has reduced contribution to the crawler movement, as it inflates less, for less time. Therefore, if one decides to increase *X* in the valve connected to the left‐hand side actuator, the crawler steers to the left. This is because the remaining actuator becomes dominant and the resultant force direction is biased.

The experiment is depicted in Figure 4C, where the output waveforms for the right and left actuators are reported with different colors, and the non‐connected third output waveform is reported in grey. As explained above the crawler is able to move forward at a speed of ≈ 0.27 BL s^−1^, as well as steering to the left and to the right (see Movie S5, Supporting Information), by manipulating the respective *X* in the circuit. Please note that inaccuracies in the manufacturing of the bellows actuators lead to a slight bias in the locomotion, visible in a small drift toward the left when the two valves are set on the same *X* (see Figure 4C).

### 2.5 Electronic‐Free Volume Controlled Fluidic Pump

In this section, we present an electronic‐free, volume‐controlled pump for microfluidics. A microfluidic pump consists of a pressure controller that can pressurize a reservoir containing a liquid and a submerged tube with an inner diameter in the scale of micrometers. By controlling the pressure and the time, these devices are able to dispense a certain amount of fluid which is then directed to a microfluidic reactor.^[^ ^35^ ^]^

**Figure 5:**
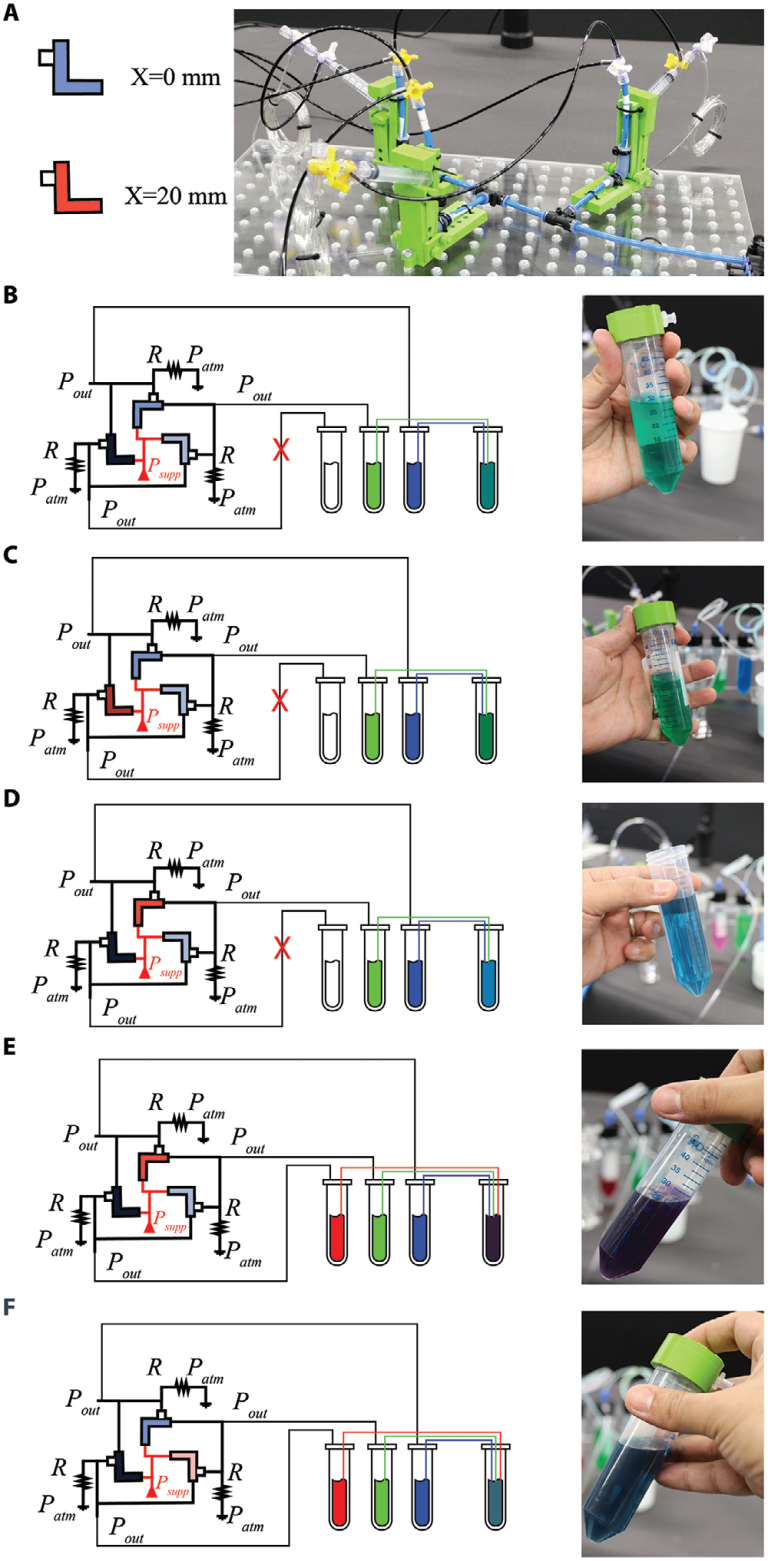
Electronic‐free volume‐controlled pump. Live photo of the ring oscillator setup and the color coding of the valves for different *X* values A). Examples of the output obtained by mixing different initial colors at different volumes guided by different ring oscillator configurations B–F).

Here, we take advantage of our reconfigurable ring oscillator (**Figure** **5**A) and its ability to have pressure outputs with tunable ON periods, to control amounts of liquids dispensed and mixed in a receiving reservoir. In order to visually distinguish between different mixtures, we used color pigments in the initial pressurized vessels, which turn into a resulting color in the receiving reservoir, depending on the configuration of the valves of the ring oscillator.

We start by connecting each of the three oscillatory outputs of the ring oscillator (θ = 55°, *P*_*supp*_ = 2 bars) to a different reservoir via a tube with an inner diameter of ${\frac{1}{32}}^{\prime \prime }$. Each tube is submerged in the pigment. Additionally, we have a second tube coming out from each reservoir, which feeds into an output reservoir, collecting the liquid mixture. At first, we used only 2 of the oscillator outputs to mix green and blue colors. Having equal actuator offsets for both active valves (Figure 5B), leads to an equal amount of color dispensed by both. As expected, we get a teal color (RGB (0, 124, 124)) in the output reservoir. As we change the *X* of the valve attached to the blue pigment to be 20 mm, the amount of blue color dispensed drops as its ON time is reduced from ≈ 4 to ≈ 2 s, giving a greener shade of teal (Figure 5C). The new color has RGB components of (4, 142, 86). This proves that a higher amount of green liquid has been dispensed compared to the blue one. When we reverse this operation we obtain a shade of teal which tends to be closer to blue. The RGB analysis of this last color in Figure 5D is (28,94,138).

As mixing pigments is a subtractive process, adding red to a mixture often results in a dark muddy brown color difficult to differentiate from black (differently from mixing light, where a combination of red, green and blue leads to white). For clarity, we hereby focus on showing different shades of colors when suppressing one of the three pigments. In Figure 5E, suppressing the green color leads to one of the Magenta color shades similar to the Blackcurrant color which when analyzed gives RGB (52,35,57). Differently, suppressing the red pigment results in one of the greenish‐blue shades color RGB (62, 102, 118), in agreement with our circuit's setup (Figure 5F).

## 3 Discussion

Here we presented a reconfigurable valve, whose switching behavior can be modified by changing one of its geometric parameters without swapping or changing any of its components. The design is programmable through a modeled design space which can guide the fabrication process toward a specific switching threshold. The valve fabrication is based on 3D printing and silicone casting, making it inexpensive and customizable. The valve working principle can be implemented in a hybrid or a completely soft design. We built a fully soft version of the presented valve (see Figure S6A,B,C) and explored its performance in a relaxation oscillator circuit (see Figure S6D,E,F, Supporting Information). This version of the valve could operate in tethered and untethered (Figure S6G, Supporting Information) circuits and exhibited high frequencies (>4 Hz, see Figure S6H,I, Supporting Information). A hybrid rigid‐soft approach allows operating at high pressures of up to 3 bars (one of the highest among all reported soft pneumatic valves), whereas a fully soft version is more compliant, can be miniaturized and achieves higher oscillation frequencies at the cost of lower operating pressures.

By splitting the soft part of the valve into two components (i.e., the bending sleeve and the pipes) we provided new insights about the importance of the cutting angle for the pipes, which in turn guides the kinking behavior of the sleeve, making it more controllable and programmable. Additionally, we show the advantage of using the sleeve's elasticity to reset the valve passively. Coupling this sleeve with an adjustable parameter (*X*), equipped our valve with on‐the‐fly reconfigurability which showed to be a valuable feature in the showcased applications.

Our analysis shows that the valve‐switching threshold is coupled to these geometric parameters. This behavior remained prominent at different supply pressures and different flow rates. It is worth noticing that this direct relationship becomes more linear at higher supply pressures as presented in the model in the Supplementary Information (see Figure S3, Supporting Information). Two parameters were kept constant in this work: the material of the soft sleeve and the linear actuator used. These might have important effects on the valve operation and will be investigated in further studies. The softer the material, the less resistant it becomes to deformation, which results in easier kinking (i.e., lower pressure threshold). Similarly, for the actuator employed in the design, a larger cross section (i.e., for the syringe piston or the pouch), exerts more force, which results in a lower pressure threshold for switching. As the design space is large, such devices can be customized for a range of applications.

Besides characterizing the behavior of our valve, we also demonstrate the advantages of introducing reconfigurability in electronic‐free components, which results in new pathways to enrich functionality and control of oscillatory circuits. For this reason, we selected two popular oscillator circuits that have recently been employed in soft robotics ‐ a relaxation oscillator and a ring oscillator. We showed how varying a simple geometrical parameter allowed us to continuously control the oscillatory frequency, spanning ten times its range. Moreover, the selective mismatching of the valves in ring oscillator circuits showed a wide spectrum of possibilities that can be harnessed for different functionalities. We believe that mismatching valves in pneumatic circuits have not been considered previously as a potential for enriching the functionality of soft robotics control circuits. This feature ‐reconfigurability‐ enabled the realization of i) a novel soft hopper, which can perform consistent hops at variable frequencies; ii) a crawler that can harness different oscillatory outputs to navigate in three directions; iii) a volume‐controlled pump that can achieve mixing of solutions with no electronic components.

Design of new electronic‐free control systems offers many advantages as demonstrated in previous studies.^[^ ^12^, ^19^, ^22^, ^23^, ^34^ ^]^ Additionally, analog control circuits and frequency‐controlled oscillators have been specifically addressed in multiple prior works. In Ref. [28] a pressure‐controlled oscillator was designed based on classical electronic voltage‐controlled oscillators. This circuit is the only work that can control the oscillating frequency of a fluidic circuit by changing a quasi‐static pressure input, alongside a constant supply pressure. However, our study is fundamentally different, as we demonstrated that our circuits can shift behavior without changing pressure input or any of the circuit components (resistors, capacitors, etc). This results in constant pressure amplitude, which is crucial when controlling soft actuators and prevents them from being damaged.

Further, in Ref. [27], the authors move one step closer to analog control of soft robots using valves. The valve they introduced has a rich design space and can be tuned in the assembly phase by changing its elastic stiffness constants. However, once manufactured, the parameters are fixed and cannot be changed during operation, making the valve's operating profiles not modifiable on‐the‐fly. On the other hand, in our design, a geometric parameter is changed by sliding one component over another. This is non‐invasive to the system and does not require changing/swapping components. Other examples have harnessed a modular approach to design wearable textile‐based soft valves,^[^ ^36^ ^]^ or software tools that utilize three different configurations of the same valve, AND, OR, and NOT (by altering the wiring of the valve ports), to optimize pneumatic circuit logic gates.^[^ ^37^ ^]^ In contrast, we here focus on reconfiguring the core component of the circuit ‐ the valve ‐ instead of relying on modularity.

The customizability and reconfigurability of our design come at a cost. For example, an important drawback is that the numerous number of components increases the chance of faults in the assembly process. Additionally, the incorrect orientation of the angled‐cut tube in the assembly can hinder the functioning of the device. For this reason, in the future, we aim to build a reconfigurable monolithic version of the valve to facilitate the fabrication process. Further, we will work on miniaturizing the device even more, as this is knowingly one of the main obstacles in the wide‐scale adoption of fluidic control devices for soft robotics.

Our work contributes to the advancement of soft robotics and their controls, harnessing the principles of reconfigurability. We believe that designing components that are reconfigurable, adaptive, and responsive to surroundings, is the way forward. In the future, this will increasingly enable the embedding of mechanical intelligence into fluidic control circuits while maintaining a minimal number of needed control inputs to the system.

## 4 Experimental Section

### Soft Inextensible Sleeve

The valve's soft sleeve was cast using DragonSkin 30 silicone rubber from SmoothOn (see Movie S1, Supporting Information). First, the inner tube of the sleeve was cast using a 3D‐printed mold in Figure S7 (Supporting Information). Then an inextensible fabric (dried PalTex wipes) was rolled around the fabricated tube. Afterward, the outer layer of the soft sleeve was cast around the fabric (see Figure S8, Supporting Information). The final result was a tube with a fabric embedded midway between the inner and the outer walls of the sleeve. This sleeve has an outer diameter of 12 mm, an inner diameter of 4.5 mm, and a length of 50 mm. The outer diameter of the sleeve affected the valve operation, as it was directly proportional with *P*_*c*_. by trial and error it was found that 12 mm is the optimal value among the tested values (11.5, 12, and 12.5 mm).

### Reconfigurable Valve

After casting the soft sleeve, it was fitted with an inlet and an outlet tube (Festo PU‐6mm OD, 4mm ID). The inlet tube was cut perpendicular to its axis and fitted 2 cm inside the sleeve. The outlet tube was cut at an angle of 45°, 55°, or 65° to the axis perpendicular to the tube's axis. A tube holder was designed and 3D printed to hold the tube in place and guide the cutting. This allowed cutting with a surgical knife and prevented tube deformation during cutting. Please note that cutting the tube with a standard pillar cutter leads to deformation of the tube before making the cut, (see Figure S11, Supporting Information), changing the orientation of the inner diameter profile compared to the outer diameter, which showed to affect the valve operation.

The design of the reconfigurable valve comprises a stationary part, a movable actuator holder, a lever, and a pin, as shown in Figure 1B. All parts were printed with an Ultimaker S5 using Ultimaker PLA filament. A commercial syringe (5 mL, RS Pro UK) was used as the valve actuator (see Movie S2, Supporting Information).

### Characterization Experiment

The characterization experiment was set up using the reconfigurable valve, a pressure controller (NI ElveFlow OB1 MK4), a large pneumatic resistor connected to the valve outlet (30 m FESTO PU tube with an inner diameter of 2 mm), a second resistor added to the actuator side (2 m FESTO PU tube with an inner diameter of 2 mm). Two distinct supply pressure sources were used. The first provides the *P*_*supp*_ for the valve, and the second supplies the pressure controller which in turn changes *P*_*in*_ following a pre‐defined profile (Figure S9, Supporting Information). The two pressure sources are separated so that *P*_*in*_ does not affect the pressure and flow rate at the valve's inlet. Two pressure sensors Panasonic ADP5150 were used to record the *P*_*in*_ and *P*_*out*_ values plotted in Figure 1E.

### Soft Hopper Fabrication

The soft bellow actuator used to build the soft hopper (Figure S5, Supporting Information) was cast using DragonSkin 30 (Smooth‐On). Two molds and cores were printed to cast two halves of the bellow actuator. Then these two parts were joined together using the same silicone rubber material. A holder for the bellow actuator was printed with access to pneumatic tubing that connects it to the relaxation oscillator circuit. The pivot of the mechanism was 3D printed and an SKF ball bearing was used to allow rotation and connect the actuator to the seesaw links (RS Pro Lead Screw 10 mm). The fluidic resistor added is a 50 cm FESTO PU tube of inner diameter 2 mm, and the volume of the actuator inner cavity is 105.37*cm* ^3^.

## Conflict of Interest

The authors declare no conflict of interest.

## Supporting information

Supporting Information

Supplemental Movie 1

Supplemental Movie 2

Supplemental Movie 3

Supplemental Movie 4

Supplemental Movie 5

## Data Availability

The data that support the findings of this study are available from the corresponding author upon reasonable request.

## References

[1] G. M. Whitesides , Angew. Chem., Int. Ed. 2018, 57, 4258. 10.1002/anie.201800907 29517838

[2] C. Laschi , B. Mazzolai , M. Cianchetti , Sci. Rob. 2016, 1, eaah3690. 10.1126/scirobotics.aah3690 33157856

[3] R. K. Katzschmann , J. DelPreto , R. MacCurdy , D. Rus , Sci. Rob. 2018, 3, eaar3449. 10.1126/scirobotics.aar3449 33141748

[4] M. Baumgartner , F. Hartmann , M. Drack , D. Preninger , D. Wirthl , R. Gerstmayr , L. Lehner , G. Mao , R. Pruckner , S. Demchyshyn , L. Reiter , M. Strobel , T. Stockinger , D. Schiller , S. Kimeswenger , F. Greibich , G. Buchberger , E. Bradt , S. Hild , S. Bauer , M Kaltenbrunner , Nat. Mater. 2020, 19, 1102. 32541932 10.1038/s41563-020-0699-3

[5] O. D. Yirmibeşoğlu , T. Oshiro , G. Olson , C. Palmer , Y. Mengüç , Front. Robot. AI 2019, 6, 40. 33501056 10.3389/frobt.2019.00040 PMC7805716

[6] J. K. Choe , J. Kim , H. Song , J. Bae , J. Kim , Nat. Commun. 2023, 14, 3942. 37402707 10.1038/s41467-023-39691-z PMC10319868

[7] M. Wehner , R. L. Truby , D. J. Fitzgerald , B. Mosadegh , G. M. Whitesides , J. A. Lewis , R. J. Wood , Nature 2016, 536, 451. 27558065 10.1038/nature19100

[8] J. Xiong , J. Chen , P. S. Lee , Adv. Mater. 2021, 33, 2002640. 33025662 10.1002/adma.202002640 PMC11468729

[9] A. T. Asbeck , S. M. De Rossi , I. Galiana , Y. Ding , C. J. Walsh , IEEE Robot. Autom. Mag. 2014, 21, 22.

[10] M. A. Robertson , J. Paik , Sci. Rob. 2017, 2, eaan6357. 10.1126/scirobotics.aan6357 33157853

[11] L. Jin , A. E. Forte , B. Deng , A. Rafsanjani , K. Bertoldi , Adv. Mater. 2020, 32, 2001863. 10.1002/adma.202001863 32627259

[12] W.‐K. Lee , D. J. Preston , M. P. Nemitz , A. Nagarkar , A. K. MacKeith , B. Gorissen , N. Vasios , V. Sanchez , K. Bertoldi , L. Mahadevan , G. M. Whitesides , Sci. Rob. 2022, 7, eabg5812. 10.1126/scirobotics.abg5812 35138883

[13] G. Gu , J. Zou , R. Zhao , X. Zhao , X. Zhu , Sci. Rob. 2018, 3, eaat2874. 10.1126/scirobotics.aat2874 33141690

[14] C. A. Aubin , R. H. Heisser , O. Peretz , J. Timko , J. Lo , E. F. Helbling , S. Sobhani , A. D. Gat , R. F. Shepherd , Science 2023, 381, 1212. 37708265 10.1126/science.adg5067

[15] D. Drotman , S. Jadhav , D. Sharp , C. Chan , M. T. Tolley , Sci. Rob. 2021, 6, eaay2627. 10.1126/scirobotics.aay2627 34043527

[16] S. Xu , C. M. Nunez , M. Souri , R. J. Wood , Sci. Rob. 2023, 8, eadd4649. 10.1126/scirobotics.add4649 37343077

[17] V. Cacucciolo , J. Shintake , Y. Kuwajima , S. Maeda , D. Floreano , H. Shea , Nature 2019, 572, 516. 31413364 10.1038/s41586-019-1479-6

[18] R. S. Diteesawat , T. Helps , M. Taghavi , J. Rossiter , Sci. Rob. 2021, 6, eabc3721. 10.1126/scirobotics.abc3721 34043529

[19] Y. Zhai , A. De Boer , J. Yan , B. Shih , M. Faber , J. Speros , R. Gupta , M. T. Tolley , Sci. Rob. 2023, 8, eadg3792. 10.1126/scirobotics.adg3792 37343076

[20] S. T. Mahon , A. Buchoux , M. E. Sayed , L. Teng , A. A. Stokes , in 2nd IEEE international conference on soft robotics (RoboSoft) , IEEE, New York 2019, pp. 782–787.

[21] K. Luo , P. Rothemund , G. M. Whitesides , Z. Suo , J. Mech. Phys. Solids 2019, 131, 230.

[22] P. Rothemund , A. Ainla , L. Belding , D. J. Preston , S. Kurihara , Z. Suo , G. M. Whitesides , Sci. Rob. 2018, 3, eaar7986. 10.1126/scirobotics.aar7986 33141749

[23] D. J. Preston , P. Rothemund , H. J. Jiang , M. P. Nemitz , J. Rawson , Z. Suo , G. M. Whitesides , Proc. Natl. Acad. Sci. USA 2019, 116, 7750. 30923120 10.1073/pnas.1820672116 PMC6475414

[24] S. Wang , L. He , P. Maiolino , IEEE Robot. Autom. Lett. 2022, 7, 3412.

[25] L. Jin , A. E. Forte , K. Bertoldi , Adv. Sci. 2021, 8, 2101941. 10.1002/advs.202101941 PMC8564437 34494725

[26] P. N. Duncan , T. V. Nguyen , E. E. Hui , Proc. Natl. Acad. Sci. USA 2013, 110, 18104. 24145429 10.1073/pnas.1310254110 PMC3831476

[27] C. J. Decker , H. J. Jiang , M. P. Nemitz , S. E. Root , A. Rajappan , J. T. Alvarez , J. Tracz , L. Wille , D. J. Preston , G. M. Whitesides , Proc. Natl. Acad. Sci. USA 2022, 119, e2205922119. 36161907 10.1073/pnas.2205922119 PMC9546565

[28] S. Song , S. Joshi , J. Paik , Adv. Sci. 2021, 8, 2100924. 10.1002/advs.202100924 PMC8529426 34459157

[29] M. Garrad , G. Soter , A. Conn , H. Hauser , J. Rossiter , Sci. Rob. 2019, 4, eaaw6060. 10.1126/scirobotics.aaw6060 33137781

[30] E. W. Hawkes , C. Majidi , M. T. Tolley , Sci. Rob. 2021, 6, eabg6049. 10.1126/scirobotics.abg6049 34043571

[31] A. A. Stanley , E. S. Roby , S. J. Keller , Sci. Adv. 2024, 10, eadl3014. 38569043 10.1126/sciadv.adl3014 PMC10990265

[32] D. W. Haldane , M. M. Plecnik , J. K. Yim , R. S. Fearing , Sci. Rob. 2016, 1, eaag2048. 10.1126/scirobotics.aag2048 33157854

[33] J. K. Yim , R. S. Fearing , in IEEE/RSJ international conference on intelligent robots and systems (IROS) , IEEE, New York 2018, pp. 2229–2236.

[34] D. J. Preston , H. J. Jiang , V. Sanchez , P. Rothemund , J. Rawson , M. P. Nemitz , W.‐K. Lee , Z. Suo , C. J. Walsh , G. M. Whitesides , Sci. Rob. 2019, 4, eaaw5496. 10.1126/scirobotics.aaw5496 33137768

[35] E. K. Sackmann , A. L. Fulton , D. J. Beebe , Nature 2014, 507, 181. 24622198 10.1038/nature13118

[36] A. Rajappan , B. Jumet , R. A. Shveda , C. J. Decker , Z. Liu , T. F. Yap , V. Sanchez , D. J. Preston , Proc. Natl. Acad. Sci. USA 2022, 119, e2202118119. 35994641 10.1073/pnas.2202118119 PMC9436326

[37] S. V. Kendre , L. Whiteside , T. Y. Fan , J. A. Tracz , G. T. Teran , T. C. Underwood , M. E. Sayed , H. J. Jiang , A. A. Stokes , D. J. Preston , G. M. Whitesides , M. P. Nemitz , IEEE Robot. Autom. Lett. 2022, 7, 6060.

