# Supplementary material for: Frequency‐Controlled Fluidic Oscillators for Soft Robots
Source: Adv Sci (Weinh). 2024 Oct 8;11(43):2408879. doi: 10.1002/advs.202408879 (PMC11578338; doi:10.1002/advs.202408879)
Supplement: Supplementary file 1 — Supporting Information [file ADVS-11-2408879-s006.pdf]

ADVANCED SCIENCE
Open Access

Supporting Information

for *Adv. Sci.*, DOI 10.1002/advs.202408879

Frequency-Controlled Fluidic Oscillators for Soft Robots

Mostafa Mousa, Ashkan Rezanejad, Benjamin Gorissen and Antonio E. Forte**

Supplementary materials

Materials and Methods
Figures S1 to S11
Movies S1 to S5

Materials and Methods

Sleeve and valve fabrication and assembly

The reconfigurable valve is a combination of three components; a soft inextensible sleeve, a rigid valve body, and a commercially available linear actuator. The soft extensible sleeve is fabricated by casting silicone rubber in two different stages. In the first stage, the inner tube of the sleeve is cast; where the mould base, the pin that defines the inner diameter, and an interchangeable third part of the mould is used to define the inner tube outer diameter are assembled.
To avoid leakage that can occur at the junction between the mould base and the pin, glue from a glue gun is applied. SmoothOn ease-release spray is applied to the mould parts for easy disassembly after the curing process. Before adding the interchangeable part to the mould, silicone is poured and vacuumed in a vacuum oven without applying heat. After removing the bubbles from the silicone which can take up to 15 minutes, the interchangeable part is added, pushing the extra silicone upwards.
After 16 hours of curing at room temperature, the mould is disassembled and the first stage of the cast is finished. The extra silicone is cut from the top of the pin, and the pin is kept inside the tube to hold it firmly when rolling the fabric. The fabric is rolled two times the circumference of the tube and is secured by glue. The pin and the coated tube are fitted back in the mould base, and silicone is poured and vacuumed, The interchangeable part is replaced with the wider diameter one and left for the final curing for another 16 hours. Finally, the mould is disassembled and the excess silicone is cut to obtain a final length of 50 mm.
After casting the sleeve, it is fitted with two Festo pneumatic tubes. The tubes used are 6 mm wide, with an inner diameter of 4 mm. The inner diameter of the sleeve is chosen to have a slightly smaller diameter so that the sleeve has a firm grip over the tube preventing leakage. The sleeve and the tubes are pre-bent and fitted into the valve body. The valve body consists of 3 main components; the valve base, adjustable actuator holder, and a lever. The actuator holder is free to move changing the parameter X, and the lever is allowed to rotate around the pin in the valve base.

Guidelines of Valve design and fitted models

The relationship between P_c, θ, and X is modelled by fitting the experimental data to a second-order polynomial equation. As we have three different sets of data based on the supply pressure, three different fittings are performed for every set of data. The fitting is carried out through Matlab using the *fit* function. The model for P_{supp} = 50, 100, 150 kPa (see eq. S1) and its coefficients are listed (see table S1).

$$\theta = A + BP_c + CX + DP_c{}^2 + EP_cX + FX^2 \tag{S1}$$

	$P_{supp} = 50$ kPa	$P_{supp} = 100$ kPa	$P_{supp} = 150$ kPa
A	-98.32	-28.78	3.428
B	3.726	1.312	0.8829
C	3.171	3.002	-0.9384
D	-0.01303	0.002041	-0.002666
E	-0.06238	-0.03104	0.005271
F	-0.01403	-0.1106	-0.000427
RMSE	9.6461	1.8685	3.0312

Table S1: **Model coefficients and its root mean square error for three different supply pressures**

This model can be used as a guideline towards valve fabrication for future systems, where θ can be decided based upon a desired P_c. Also, the model provides an estimation on how P_c will change during operation upon sweeping X. The fitted models' plots are shown in Fig. S3.

Soft hopper and seesaw mechanism setup.

The soft hopper is a bellow extendable silicone rubber actuator. The actuator is cast from DragonSkin 30 where every half is cast at a time. The two halves are then joined together and placed on a common base using the same silicone material. The seesaw mechanism is built by 3D printing a pivot of 150 mm in length. An SKF ball bearing is fitted in a 3D-printed case that is assembled on the top of the pivot. The inner diameter of the bearing is connected to two laser-cut links by a 3D-printed setup to create a seesaw. On one side the link is attached to a counterweight, and on the other side the soft hopper is attached to the links through another SKF ball bearing. The ball bearing allows the bellow actuator to perform hopping while maintaining its vertical orientation.

Soft Miniaturised Valve

To explore the capabilities of this design principle, and to investigate its limitations, we developed a soft miniaturised version of the valve (see Fig. S6A, B, C). This version is smaller in dimensions; $70 \times 30 \times 30$ mm compared to the original version ($100 \times 90 \times 35$ mm). The syringe is replaced by a soft plastic pouch. A soft valve body is cast by PDMS to replace the 3D-printed valve body. The same soft sleeve is used, except that it has a different inner diameter (3 mm) which allows it to be used in a flat configuration (less bending is needed to block a flow).

This soft valve is used to build a relaxation oscillator(see Fig. S6D, E, F), where its outlet is connected to the plastic pouch that is connected to the outlet tube by a slider. The operating principle is similar to the relaxation oscillator previously reported in the soft hopper section, where the X parameter in this case is the distance travelled by the slider on the outlet tube. This valve could be operated untethered (see Fig. S6G) using a FESTO^{TM} 0.75 L canister and could generate high-frequency oscillations in both untethered and tethered configurations (see Fig. S6H, I).

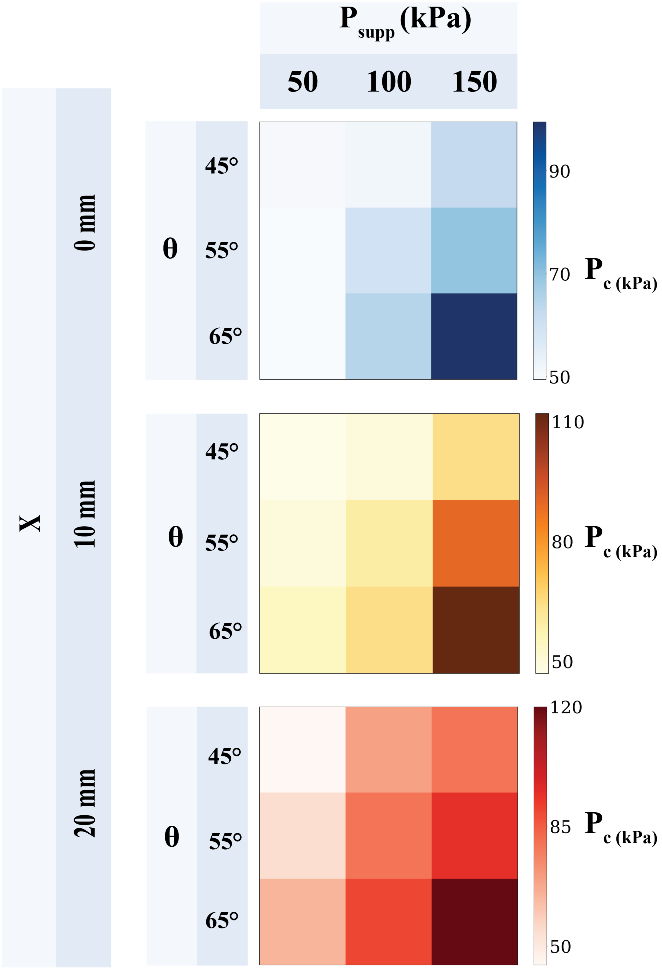

Figure S1: **Threshold pressure P_c heatmap.** A heatmap showing the distribution of the valve threshold pressure for each supply pressure when changing both θ and X

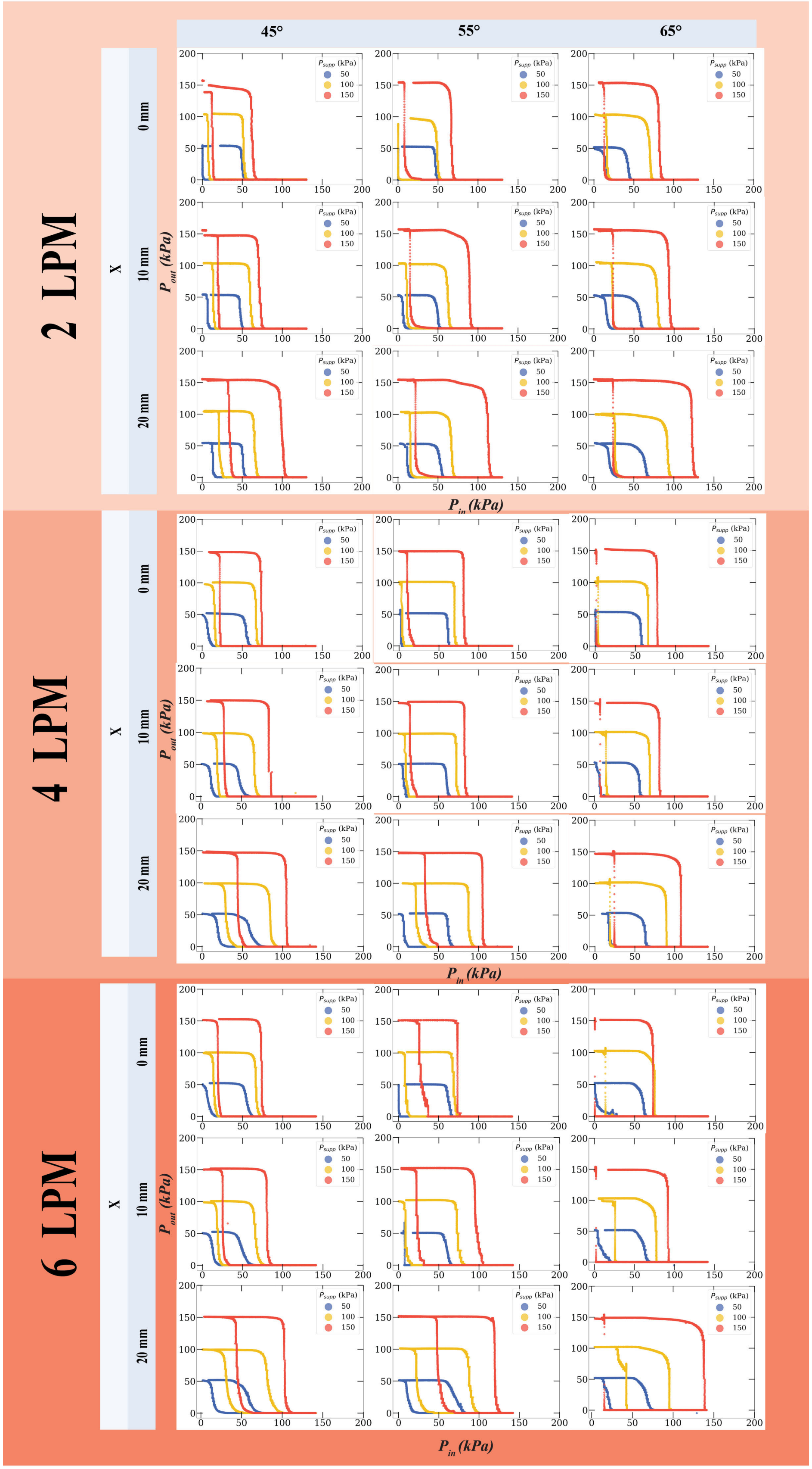

Figure S2: **Extended characterisation experiments** Valve characterisation at three different flow rates 2, 4, and 6 LPM.

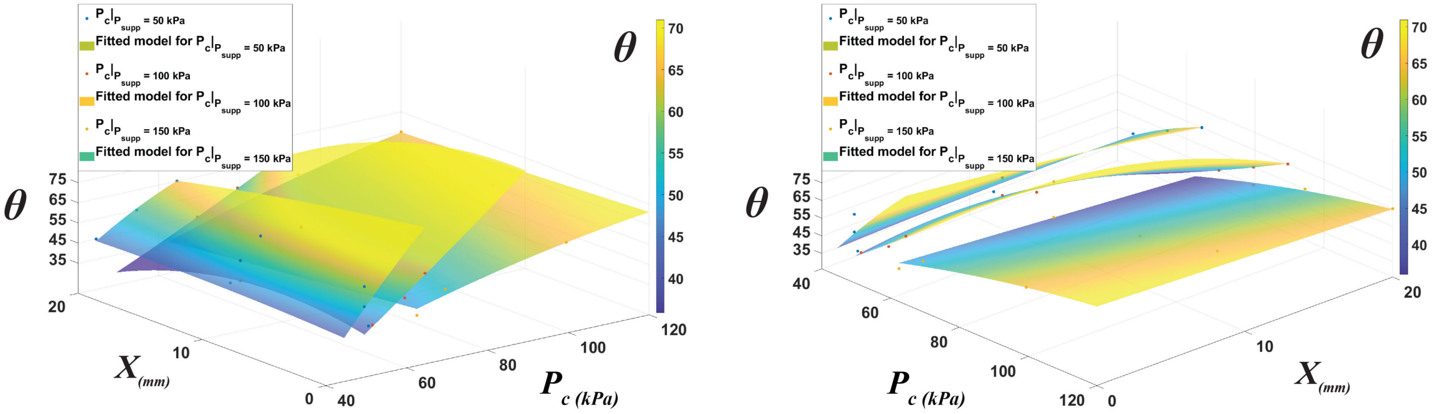

Figure S3: **Fitted models** The surfaces for fitted models for $P_{supp} = 50, 100, 150$ kPa and the data points from two different prespectives.

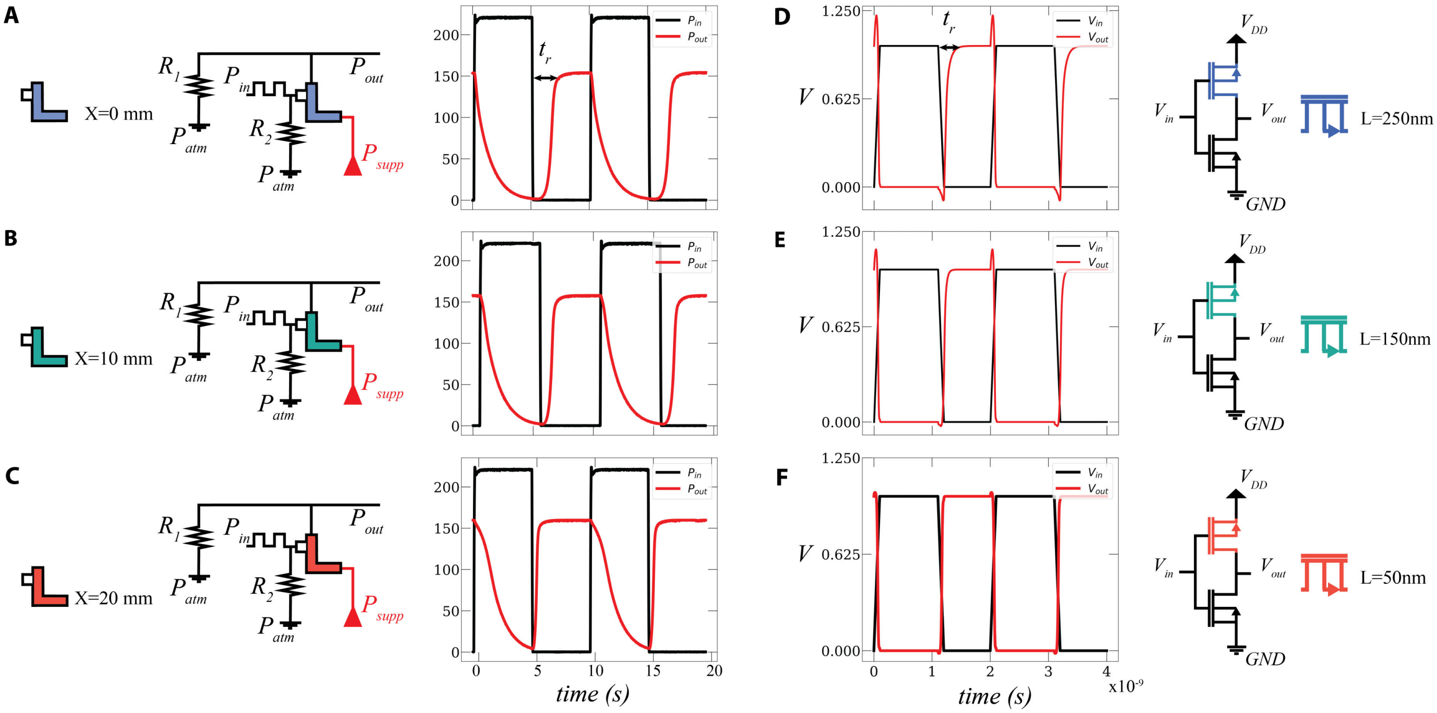

Figure S4: **Change in rise times for pneumatic and electronics inverters.** The rise time shown as t_r decreases on increasing X (A-C). The same behaviour emerges in the electronic inverter on decreasing the PMOS length (D-F).

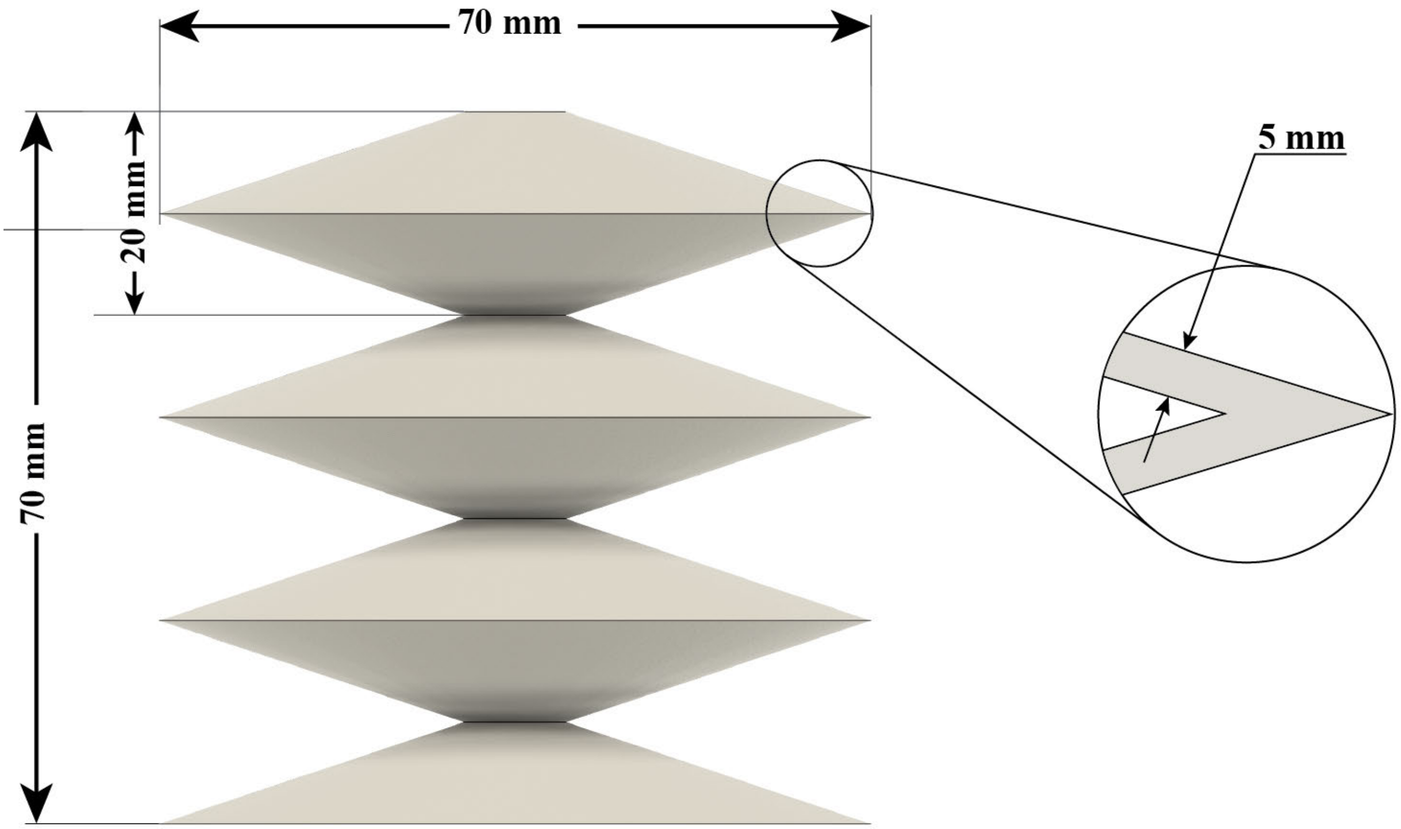

Figure S5: **The bellow actuator** The actuator used in the soft hopper application has a full length of 70 mm and cross-section thickness of 5 mm that is constant throughout the actuator

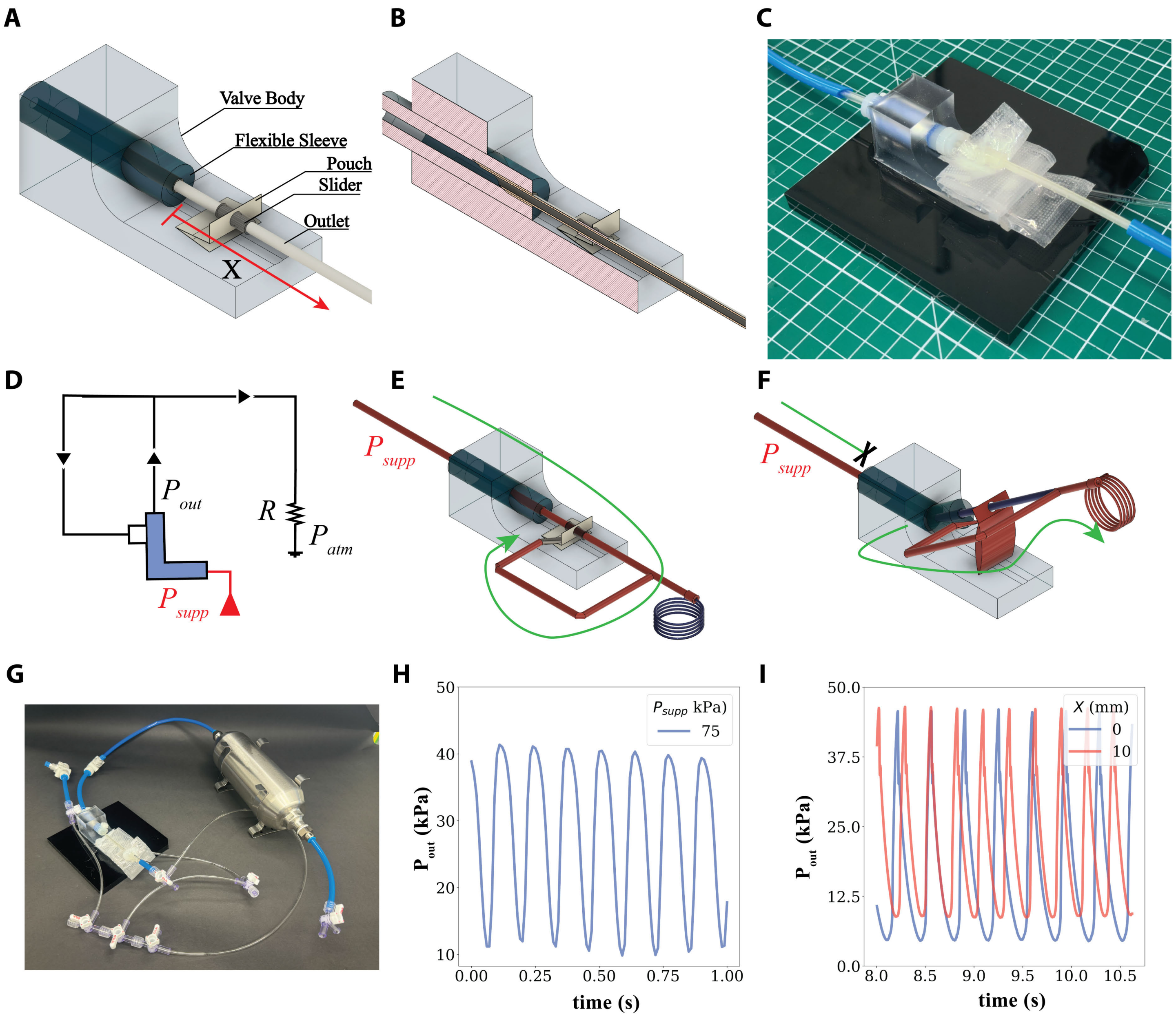

Figure S6: **Soft Miniaturised Valve** The components of the soft valve (A) and a section view showing the assemblage (B). Live photo of the soft valve (C). Illustration of the soft relaxation oscillator circuit (D) with the airflow shown by the green line and the pressurised regions highlighted in red during the charging (E) and discharging phase (F). Live photo of the untethered circuit (G) and the output waveforms of the untethered (H) and tethered (I) oscillations

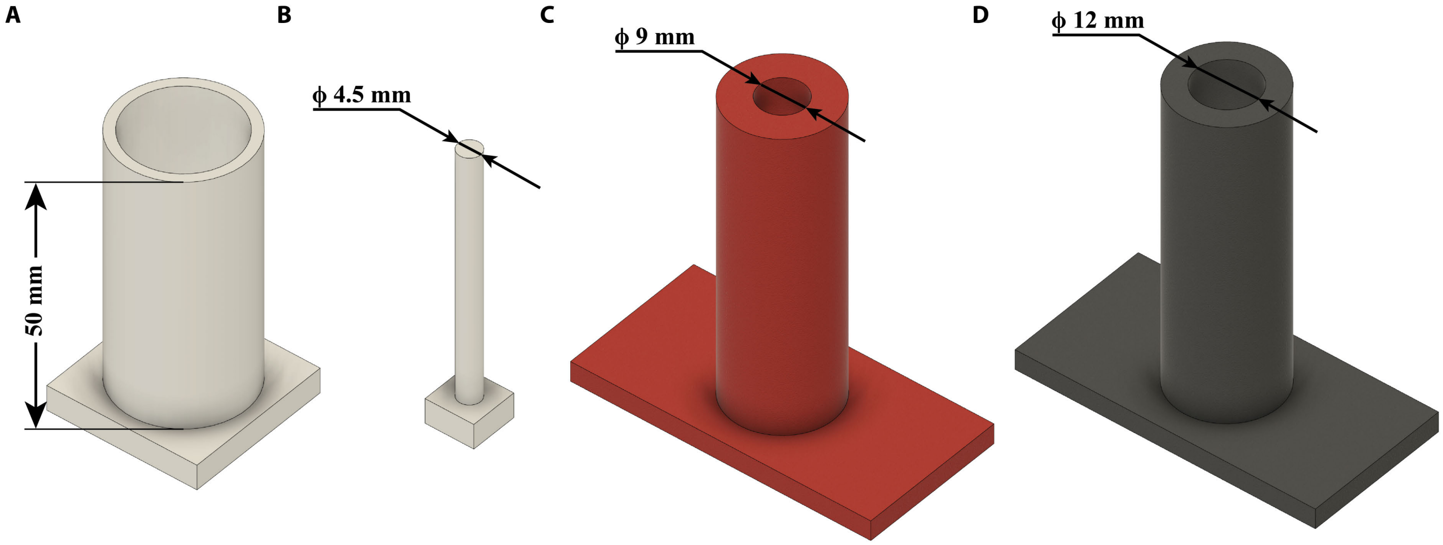

Figure S7: **The mould for flexible sleeve** The main body of the mould with a height of 50 mm (A). The pin which defines the sleeve inner diameter (4.5 mm) (B). Two interchangeable parts (C) and (D), are used in the first and second stages of casting, respectively.

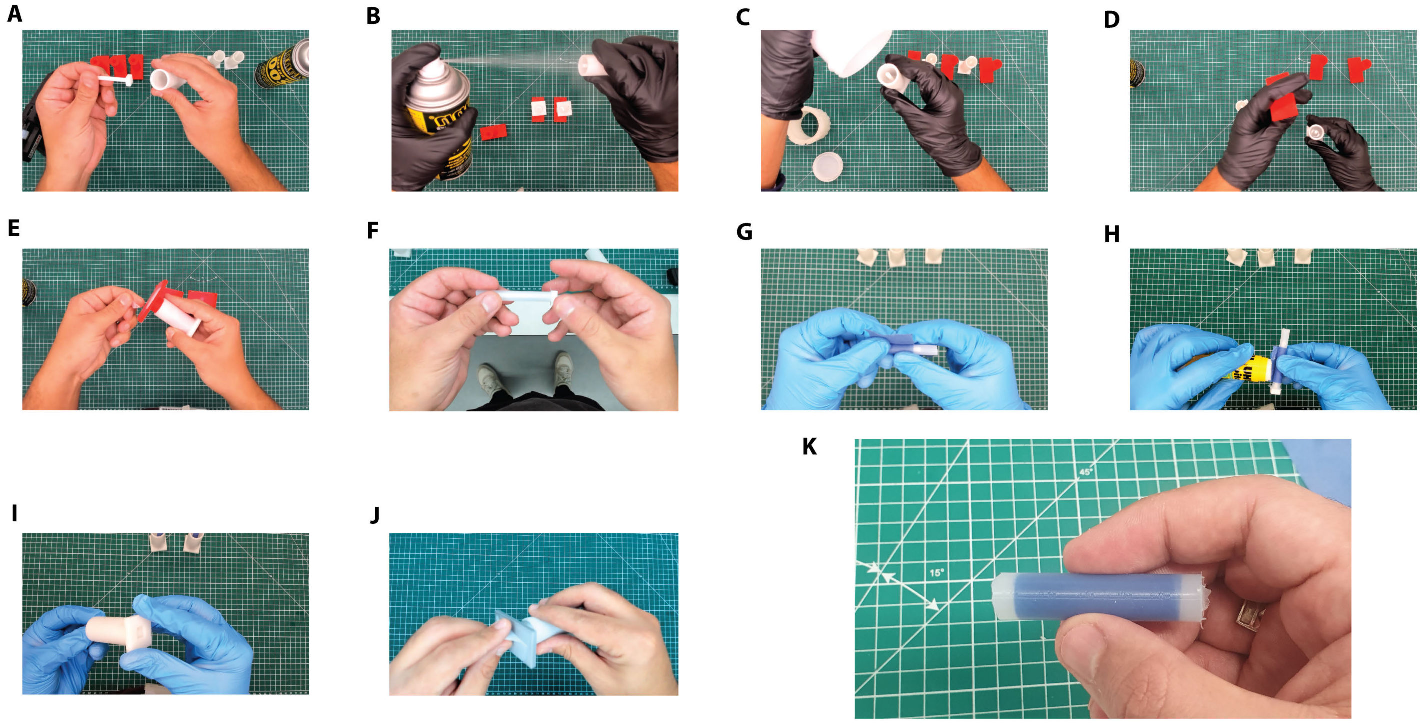

Figure S8: **Soft sleeve fabrication.** The 3D printed mould assembly (A). Applying ease-release spray to the assembled mould (B). Adding mixed silicone rubber to the mould (C). After vacuuming the silicone, adding the last part of the mould that defines the diameter of the sleeve's inner tube (D). After curing, disassembly of the mould (E). The inner tube shape after curing (F). Rolling the fabric around the sleeve's inner tube (G), and securing it with glue (H). Fitting it back in the mould (I), using a wider diameter mould part and repeating the casting steps. Final disassembly of the mould (J) and the final sleeve (K).

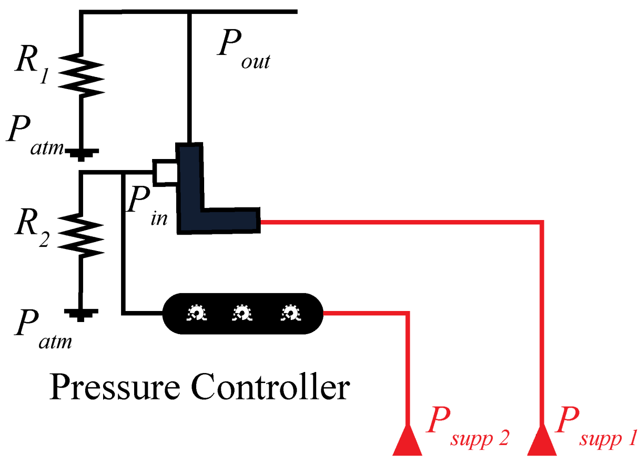

Figure S9: **Valve characterisation experiment setup.** Two supply sources are used; one to supply the pressure controller that controls P_{in}, the other is used as P_{supp}, which is switched on/off by the valve. The output of the valve and the valve actuator both are connected to a pull-down resistor to the atmosphere.

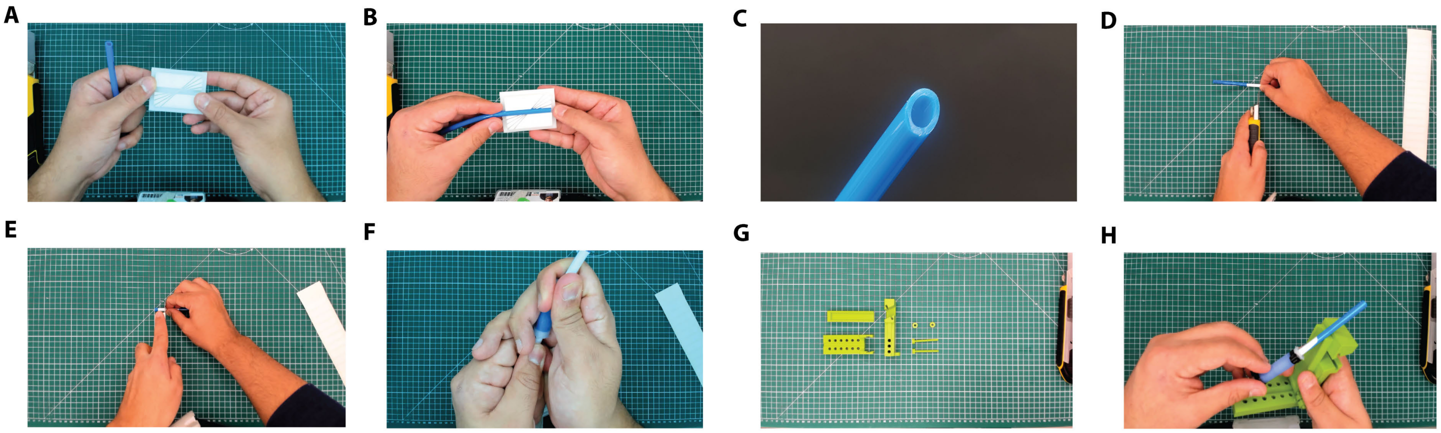

Figure S10: **Valve assembly process.** The tube cutting platform that prevents the tube from deforming during cutting (A). Securing and cutting the tube in the platform (B), results in a cut with a uniform profile (C). Marking the inlet (D) and outlet (E) tubes at the lengths to be inserted inside the sleeve (F). Valve components are then assembled (G) and the sleeve with tubes are fixed to the valve body using a zip tie (H)

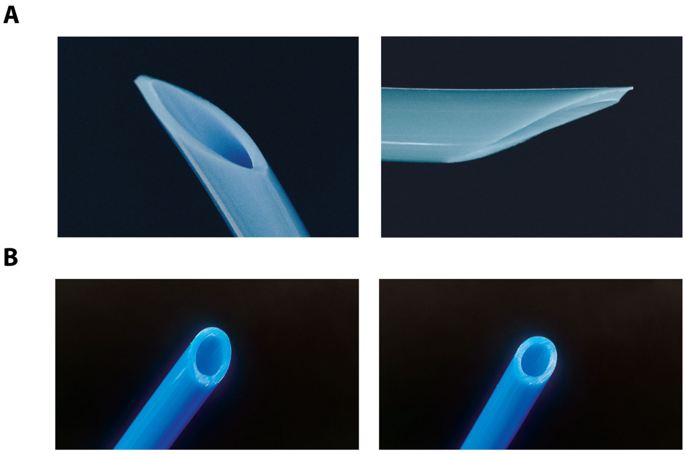

Figure S11: **Importance of cutting method.** The tubes cut with a standard cutting pillar show a profile of the inner diameter which is not in the same plane as the outer diameter (A). Instead, our method guarantees that both inner and outer diameter profiles lay on the same plane, ensuring complete obstruction of the airflow when the sleeve is kinked.
